# A Cell-Free SDKP-Conjugated Self-Assembling Peptide Hydrogel Sufficient for Improvement of Myocardial Infarction

**DOI:** 10.3390/biom10020205

**Published:** 2020-01-30

**Authors:** Saman Firoozi, Sara Pahlavan, Mohammad-Hossein Ghanian, Shahram Rabbani, Shima Tavakol, Maryam Barekat, Saeed Yakhkeshi, Elena Mahmoudi, Mansoureh Soleymani, Hossein Baharvand

**Affiliations:** 1 Department of Tissue Engineering and Regenerative Medicine, Faculty of Advanced Technologies in Medicine, Iran University of Medical Sciences, Tehran 1449614535, Iran; saman.nano1989@gmail.com; 2 Department of Stem Cells and Developmental Biology, Cell Science Research Center, Royan Institute for Stem Cell Biology and Technology, ACECR, Tehran 1665659911, Iran; sarah.pahlevan2007@gmail.com (S.P.); saeedyakhkeshi@gmail.com (S.Y.); 3 Department of Cell Engineering, Cell Science Research Center, Royan Institute for Stem Cell Biology and Technology, ACECR, Tehran 1665659911, Iran; biomaterialist@gmail.com; 4 Research Center for Advanced Technologies in Cardiovascular Medicine, Tehran Heart Center, Tehran University of Medical Sciences, Tehran 1416753955, Iran; sh-rabbani@tums.ac.ir; 5 Cellular and Molecular Research Center, Iran University of Medical Sciences, Tehran 1449614535, Iran; shima.tavakol@yahoo.com; 6 Department of Regenerative Medicine, Cell Science Research Center, Royan Institute for Stem Cell Biology and Technology, ACECR, Tehran 1665659911, Iran; barekat1001@yahoo.com; 7 Massachusetts General Hospital, Harvard Medical School, Boston, MA 02114, USA; elena.mahmoudi@gmail.com; 8 Department of Developmental Biology, University of Science and Culture, ACECR, Tehran 1461968151, Iran

**Keywords:** mesenchymal stem cells, cardiac cell therapy, self-assembling peptide hydrogel, myocardial infarction

## Abstract

Biomaterials in conjunction with stem cell therapy have recently attracted attention as a new therapeutic approach for myocardial infarction (MI), with the aim to solve the delivery challenges that exist with transplanted cells. Self-assembling peptide (SAP) hydrogels comprise a promising class of synthetic biomaterials with cardiac-compatible properties such as mild gelation, injectability, rehealing ability, and potential for sequence modification. Herein, we developed an SAP hydrogel composed of a self-assembling gel-forming core sequence (RADA) modified with SDKP motif with pro-angiogenic and anti-fibrotic activity to be used as a cardioprotective scaffold. The RADA-SDKP hydrogel was intramyocardially injected into the infarct border zone of a rat model of MI induced by left anterior descending artery (LAD) ligation as a cell-free or a cell-delivering scaffold for bone marrow mesenchymal stem cells (BM-MSCs). The left ventricular ejection fraction (LVEF) was markedly improved after transplantation of either free hydrogel or cell-laden hydrogel. This cardiac functional repair coincided very well with substantially lower fibrotic tissue formation, expanded microvasculature, and lower inflammatory response in the infarct area. Interestingly, BM-MSCs alone or in combination with hydrogel could not surpass the cardiac repair effects of the SDKP-modified SAP hydrogel. Taken together, we suggest that the RADA-SDKP hydrogel can be a promising cell-free construct that has the capability for functional restoration in the instances of acute myocardial infarction (AMI) that might minimize the safety concerns of cardiac cell therapy and facilitate clinical extrapolation.

## 1. Introduction

Myocardial infarction (MI) is the most prevalent among cardiac disorders and has high morbidity and mortality rates [1]. Blockage of the coronary artery results in MI that is characterized by local ischemia and apoptosis of conforming cardiac cells that include cardiac progenitor cells (CPCs), cardiomyocytes, smooth muscle cells, and endothelial cells [2]. Inflammatory cells migrate to the infarcted area and produce large amounts of reactive oxygen species (ROS) [3]. Thus, the cardiac environment converts to an inflamed area with low oxygen levels, high amounts of ROS, large numbers of apoptotic cells, and disintegrated extracellular matrix (ECM). Once inflammation is reduced, cardiac tissue is replaced by scar tissue, which is accompanied by dilation of the left ventricle (LV) [4,5]. Conventional therapeutic approaches for MI have been developed based on revascularization by different means such as balloon angioplasty or coronary artery bypass grafting [6]. Although mostly successful, these revascularization methods could not fully inhibit ventricular remodeling due to hypoxia in the infarct area. One major challenge is restoration or replacement of damaged myocardium. Injured myocardium leads to development of dilated ventricles and consequent heart failure [7]. Cardiac cell therapy is a promising approach for myocardial regeneration [8]. Various cell sources have been transplanted into the infarcted area to restore cardiac function via direct myogenesis such as CPCs [9] and mesenchymal stem cells (MSCs) [10] or their paracrine effects on resident cells such as CPCs [9], MSCs [10], endothelial progenitor cells (EPCs) [11], and hematopoietic stem cells (HSCs) [11]. In particular, several clinical trials have been designed or conducted based on the promising results of MSC therapy for MI (clinicaltrials.gov identifier: NCT03798353, NCT02323477, NCT01392105, NCT00114452, NCT01291329). However, cardiac cell therapy has shown partial success, which is likely due to low engraftment of the transplanted cells [12,13]. Both viability and retention of the transplanted cells could be hampered by insufficient nutrition caused by lack of vasculature, inflammatory conditions, and remodeled ECM in the fibrotic area [14].

To circumvent the challenges of cell therapy, tissue engineering has emerged as an option to induce cardiac repair using biomaterial scaffolds as cell delivery vehicles or cell-free therapeutic agents. With scaffold-mediated cell delivery, a higher rate of cell retention and viability could be achieved due to greater cell–ECM interactions at the transplantation site [15]. More importantly, the biomaterial scaffolds could work alone as a cell-free approach to induce cardiac repair by providing mechanical support for the dilated ventricle and/or release of bioactive agents to induce angiogenesis, cell recruitment, and immunomodulatory effects [16]. Hydrogels are a subgroup of biomaterials that have a high water content and tunable crosslink density, which allows for controlled delivery of cells and other bioactive agents [16,17,18]. Hydrogel-based scaffolds can be injected into the infarcted site via minimally invasive surgical methods. Thus, they are of special interest as a therapeutic approach to prevent adverse LV remodeling [19]. A vast pool of materials have been assessed for this purpose, and range from natural polymers such as myocardial ECM [20], hyaluronate [21], collagen [22], fibrin [23], and alginate [24] to synthetic polymers such as ureido-pyrimidinone-modified poly(ethylene glycol) [25] and poly(*N*-isopropylacrylamide) [26]. Despite a wide range of preclinical studies with promising outcomes, their translation to the clinic has been limited to a very few hydrogels that have reached final phases of clinical trials. These include myocardial ECM hydrogel (clinicaltrials.gov identifier NCT02305602) and alginate hydrogel (NCT3082508 and NCT01311791). Reasons for this unsuccessful translation include safety concerns and batch-to-batch variations of the natural polymers, whereas the alternative synthetic polymers usually suffer from poor bioactivity [27]. Self-assembling peptides (SAPs) are a class of synthetic polymers that can be tailored to form bioactive hydrogels through biocompatible supramolecular interactions. SAPs are composed of a series of hydrophobic and hydrophilic amino acids that can be induced for structural organization by the addition of multivalent ions [28]. SAP hydrogels have been used to treat MI in preclinical studies with promising outcomes [29,30,31]. In one study, an SAP hydrogel with heparin-binding peptide motif that was amenable to vascular endothelial growth factor (VEGF) and bFGF binding was injected at the site of injury in an acute MI model. The results showed improved hemodynamic functions [32]. Recently, an MSC-laden SAP hydrogel was used in the form of epicardial coating at the site of infarction and resulted in enhanced left ventricular ejection fraction (LVEF%) after four weeks [15].

The moldable synthesis of SAPs could allow for incorporation of bioactive peptide motifs to the main hydrogel-forming oligopeptide, which is the core motif [28]. Herein, we conjugated a bioactive motif (SDKP) with angiogenic, anti-inflammatory, and anti-fibrotic functions [33,34,35,36,37] to a common core motif composed of four RADA sequences whose final product was an injectable supramolecular hydrogel ((RADA)4-SDKP) The angiogenic potential of the (RADA)4-SDKP hydrogel was shown by in vitro and ex ovo experiments. We applied the (RADA)4-SDKP hydrogel in a rat model of MI to alleviate the inflammatory conditions and provide a permissive environment for revascularization of the damaged myocardium. These features were confirmed by the results of in vivo analysis. Interestingly, the cell-free hydrogel was highly cardioprotective, improved functional parameters and reduced scar formation at the infarct area, and was comparable with the MSC-laden hydrogel.

## 2. Materials and Methods

### 2.1. Preparation of the (RADA)4-SDKP Hydrogel

RADA16-I was selected as the core due to its well-known ability for mild gelation via self-assembly [15], GG was employed as spacer, and this construct was decorated with SDKP motif due to its biological functions. RADA16-I was selected based on the design of a commercial product named PuraMatrixTM (PuraMatrixTM, BD, Erembodegem, Belgium) [38,39] that has also been used for cardiac repair [2]. (RADA)4-SDKP peptide was custom synthesized by DG Peptides Co., Ltd. (Hangzhou, China). The (RADA)4-SDKP powder was dissolved in deionized water (DEPS) at a concentration of 0.5%. Then, it was sonicated for 30 min before the experiments. To prepare the hydrogel, we mixed the (RADA)4-SDKP solution with an equal volume of phosphate-buffered saline (PBS) to form the gel with a final concentration of 0.25% after 1 min. The 0.25% concentration of SAP hydrogel has been used before [40] and proposed by in vivo protocols of PuraMatrix^TM^, which is composed of (RADA)4 hydrogel.

### 2.2. Characterization of the (RADA)4-SDKP Hydrogel

The viscoelasticity of SAP hydrogel was analyzed using a stress-controlled rheometer (Paar Physica, MCR300 SN599139, EU). We injected 500 µL of the hydrogel into the lower plate (10 mm diameter and 1 mm thickness). The upper plate (20 mm diameter) was lowered until it contacted the gel surface, and the examination was done by oscillatory frequency sweeps (0.1–600 s^−1^, 0.5% strain). The microstructure of the SAP hydrogel was assessed by a field emission scanning electron microscope (FESEM) (MIRA3\Tescan, Brno, Czech Republic). For this purpose, 500 µl of the hydrogel were frozen at −20 °C and lyophilized for 48 h. The obtained sponge was sputter coated with gold and observed using FESEM.

### 2.3. Cytocompatibility of the (RADA)4-SDKP Hydrogel

#### 2.3.1. Isolation of Neonatal Mouse Cardiomyocytes (NMCMs)

NMCMs were isolated from one-day-old pups according to a protocol described previously [41]. All animal studies were performed in accordance with guidelines approved by the Ethics Committee of Royan Institute (Tehran, Iran IR.ACECR.ROYAN.REC.1398.192). Briefly, the hearts were excised and rapidly rinsed in cold Hank’s balanced salt solution (HBSS, Gibco USA). All non-cardiac tissues and the atria were carefully dissociated from the ventricles. Then, the ventricles were further rinsed in cold HBSS to remove any remaining blood and then they were minced into small pieces. Next, the pieces were treated with HBSS/trypsin (0.5 mg/mL) overnight at 4 °C on an orbital shaker at 80 rpm. After the predigestion step, warm culture medium that consisted of 75% Dulbecco’s Modified Eagle Medium (DMEM, Gibco), 25% Media 199 (M-199, Gibco), 1% penicillin/streptomycin (pen/strep, Gibco), 1% l-glutamine (Gibco), and 1% 4-(2-hydroxyethyl)-1-piperazine ethanesulfonic acid (HEPES, Gibco) was added to the trypsinized heart tissues. After discarding the predigestion media, the heart tissue was subjected to serial digestions with an HBSS/collagenase II (0.8 mg/mL) solution on an orbital shaker at 37 °C. Following the digestion steps, we collected the supernatants, centrifuged them at 800 rpm for 5 min at room temperature, and resuspended the cell pellet in culture media composed of DMEM, M-199, 1% pen/strep, 1% l-glutamine, 1% HEPES, 20% horse serum (Gibco), and 10% fetal bovine serum (FBS, Invitrogen, Carlsbad, CA, USA). The cell suspension was preplated on 0.1% gelatin-coated T-75 flasks for 1 h at 37 °C and 5% CO_2_ to remove the isolated cardiac fibroblasts and obtain a homogeneous population of cardiomyocytes. Finally, the preplate supernatant was collected and centrifuged, and the pellet of NMCMs was resuspended in culture media.

#### 2.3.2. MTS Assay

The impact of (RADA)4-SDKP hydrogel encapsulation on metabolic activity and proliferation of NMCMs was evaluated by MTS assay. Briefly, 1 × 10^4^ NMCMs were cultured alone (control) or encapsulated in the (RADA)4-SDKP hydrogel at a final concentration of 0.25% *v/w* into each well of a 96-well plate that contained culture media for 24, 48, and 72 h at 37 °C and 5% CO_2_ (*n* = 4). After each incubation period, the cell-seeded plates or cell-laden gels (*n* = 4) were incubated for 4 h with MTS reagent (Promega, USA) and the supernatant was analyzed for absorbance at 490 nm.

### 2.4. Angiogenic Potential of (RADA)4-SDKP Hydrogel In Vitro and Ex Ovo

#### 2.4.1. In Vitro Vascular Endothelial Growth Factor (VEGF) Secretion Assay

VEGF release by human umbilical vein endothelial cells (HUVECs) was evaluated in two forms of the cultured cells alone or encapsulated within (RADA)4-SDKP. For this purpose, HUVECs were isolated from aseptic human umbilical cords that were received from Arash Hospital (Tehran, Iran) after obtaining written consent from volunteer couples, as previously described [42]. The HUVECs were cultured in EGM-2 supplemented with 10% FBS (10,270, Gibco). All in vitro experiments were performed using passages 3–6 HUVECs, and the cells were incubated at 5% CO_2_ and 37 °C and tested regularly for mycoplasma contamination by our laboratory. Then, 1 × 10^4^ HUVECs were cultured alone (control) or encapsulated into the hydrogel at a final concentration of 0.25% *v/w* onto each well of a 48-well plate that contained the aforementioned medium for 24 or 124 h (*n* = 3). Next, conditioned media from the cultured cells were collected and assessed by enzyme-linked immunosorbent assay (ELISA) using a Human VEGF DuoSet ELISA DY293B-05 kit (R&D Systems, Minneapolis, Minnesota, USA) according to the manufacturer’s instructions.

#### 2.4.2. Chicken Chorioallantoic Membrane (CAM) Assay

Fertilized eggs from Hy-line W-36 hens were obtained from a commercial farm. The eggs were cracked under a sterile laminar flow hood and the contents were transferred to a Petri dish. Each embryo with the yolk was transferred to a surrogate shell, which was 3–4 g heavier than the egg shell, sealed with plastic wrap, and allowed to incubate in a forced-air incubator for 60 h at 37 °C and 60% humidity. The embryonic day (ED) when the eggs were placed in the incubator was considered to be embryonic day 0 (ED0). On ED2.5, the yolk-embedded embryo was transferred to a second surrogate shell, which was 35 to 40 g heavier than the egg shell, sealed with plastic wrap, and allowed to incubate for another 5 days. Dead or infected embryos were removed daily to avoid further contamination. The chorioallantoic membrane (CAM) angiogenesis assay was performed as previously described [43]. Briefly, O-ring paper filters that contained PBS (vehicle) or (RADA)4-SDKP hydrogel at a final concentration of 0.25% *v*/*w* (gel) were deposited on the intact CAMs at ED9, at a location distal from the embryo and proximal to the major blood vessels. The embryos were maintained in the incubator for 72 h. At ED12, the embryos were transferred to the stage of a SZX16 Wide Zoom Versatile Stereo Microscope (Olympus, Yokohama, Japan) and images were taken from inside the O-rings. The numbers of branches were calculated for five random images in each treatment and averaged.

### 2.5. Cardiac Repair by (RADA)4-SDKP Hydrogel

#### 2.5.1. Establishment of an Acute Myocardial Infarction (AMI) Rat Model

All animal experiments were approved by the Royan Institute Ethics Committee in accordance with the NIH Guidelines for the Care and Use of Laboratory Animals (NIH Publication No. 85e23, revised 2010). Adult male Sprague Dawley rats (280–350 g) were anesthetized with intraperitoneal (IP) injections of 0.1 mg/kg medetomidine (Laboratorios Syva, AEM, Spain) and 75 mg/kg ketamine (Alfasan, Woerden The Netherlands). To maintain a deep level of anesthesia, intubation and mechanical ventilation (Harvard, state abbreviation, USA) with a mixture of room air, oxygen, and 1% isoflurane was performed. The chest area was shaved and a left thoracotomy was performed to expose the LV. The rat model of AMI was achieved by permanent ligation of the left anterior descending artery (LAD) with 6-0 monofilament silk suture material (Keyhan Teb, TehranIran). Validity of the AMI model was evaluated by color change to pale at the vicinity of the ligation and echocardiographic measurement one day after induction. Rats with EF < 40% were selected as AMI models.

#### 2.5.2. Treatment Procedure

Human bone marrow mesenchymal stem cells (BM-MSCs) at passages 3–5 were used as the cell source. The BM-MSCs were obtained from the Cell Bank (RSCB0178) of Royan Institute for Stem Cell Biology and Technology (Tehran, Iran) whose bone marrow was taken from a 34-year-old healthy male after written consent. The cells were cultured in α-MEM with 20% inactivated FBS that contained l-glutamine, penicillin, and streptomycin. The AMI rats were randomly divided into four experimental groups: Vehicle, Cell, Gel, and Gel + Cell. The Vehicle group received an intramyocardial injection of PBS (100 µL) at four sites of the infarct border zone. The Cell group received 2 × 10⁶ human BM-MSCs in PBS (100 µL). The Gel group received 0.25% SAP hydrogel (100 µL) and the Gel + Cell group received 2 × 10⁶ BM-MSCs encapsulated in 0.25% SAP hydrogel (100 µL). Number of injected cells at Cell and Gel + Cell groups was chosen based upon previous reports [44]. After cell and/or hydrogel transplantation, each animal received an IP injection of 1 mg/kg atipamezole (Laboratorios Syva) and the animal was kept in an oxygen chamber on a heating stage until recovery. A prophylactic antibiotic ((Enrofloxacin, 15 mg/kg, Rooyan Darou, Tehran, Iran) and analgesic (tramadol, 20 mg/kg, Darou Pakhsh, Tehran, Iran) were injected IP for three days to prevent infection and pain.

#### 2.5.3. Echocardiography Measurements

Functional evaluations of the hearts were performed by echocardiography using an ultrasound system (Mindray, city, state abbreviation, Hamburg, Germany) 1 day before the AMI induction (Pre), at day 1 after the AMI (Post1), and 4 weeks after the AMI (Post2). Rats were anesthetized by IP injections of 0.1 mg/kg medetomidine and 75 mg/kg ketamine, and their chests were shaved. A cardiologist, blinded to the treated groups, evaluated the cardiac function by ultrasound. Two-dimensional images were taken to illustrate functional echocardiography parameters. End-diastolic and systolic volumes were obtained to assess the LVEF. The changes in ejection fraction from day 1 to day 28 (∆EF) were calculated and plotted. Fractional shortening (FS), the changes of fractional shortening from day 1 to day 28 (∆FS), left ventricular inner diameter at diastole (LVIDd), and left ventricular inner diameter at systole (LVIDs) were extracted and depicted. The rats were euthanized at week 4 and their hearts were harvested for histological evaluations.

#### 2.5.4. Histological Studies

On day 28, the hearts were excised and inundated in formalin 10% for 2 days. Following fixation, processing, and embedding in paraffin, the sections were cut into 5 µm increments. After deparaffinization and hydration, Masson’s trichrome (MT) staining was carried out on the heart sections. Then, percentage of fibrotic areas were determined by ImageJ software (version 1.47, acquired from http://imagej.nih.gov/ij/). The averaged infarct area was calculated from 12 slides in each animal, plotted, and compared between the experimental groups.

Sections from apex, middle, and base levels of each animal heart were chosen. Immunofluorescence staining of the deparaffinized and dehydrated sections was performed by subjecting the slides to antigen retrieval with Target Retrieval Solution (pH 9, Dako, S2368, Glostrup, Denmark) for 20 min. The sections were then permeabilized with 0.5% Triton X-100 at RT for 30 min, blocked for 1 h at 37 °C, and incubated overnight with primary antibodies α-SMA (Abcam: ab7817, Cambridge, Massachusetts, USA), CD68 (Sigma: 048982, Darmstadt, Germany), and STEM 121 (Clontech, Y40410, USA) at 4 °C. Secondary antibody (anti-mouse IgG, Sigma, F9006) was applied at 37 °C for 1 h, counterstained with DAPI, and the sections were observed under a fluorescence microscope (Olympus; BX51). To quantify vascular density, the number of α-SMA^+^ structures at whole infarct zone of each section was assessed and the average number was plotted for each animal as number of α-SMA^+^ structures/field of infarct area [45]. Similarly, the numbers of CD68⁺ cells were counted in sections from apex, middle, and base to assess infiltration amount of macrophages at the site of infarction and expressed as number of CD68⁺ cells/field of infarct area [46]. In order to track the transplanted MSCs, immunostaining of STEM121 as specific antibody against a cytoplasmic protein of human cells was performed.

### 2.6. Statistical Analysis

All data are presented as mean ± standard deviation (SD). Statistical analysis was performed using GraphPad Prism 8.0.1 software (San Diego, CA, USA) by one-way analysis of variance (ANOVA) followed by the Tukey post-hoc test for comparisons between three or four groups and the Student’s *t*-test for comparison between two groups. The survival experience in groups was analyzed by log-rank (Mantel–Cox) and Gehan–Breslow–Wilcoxon tests. *p* < 0.05 was considered statistically significant.

## 3. Results

### 3.1. Structure and Characterization of Self-Assembling Peptide (SAP) Hydrogel

**Figure 1:**
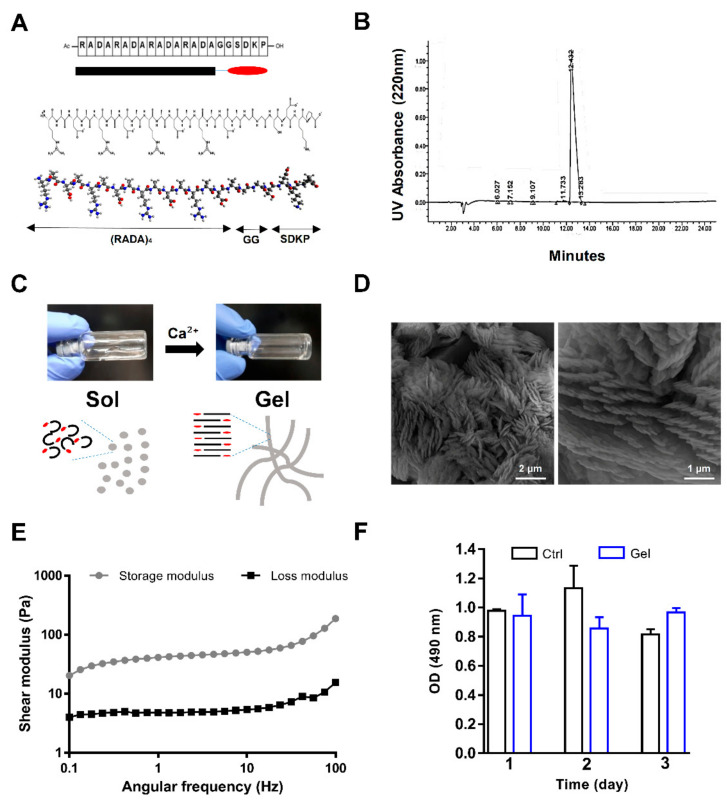
Structure and characterization of the self-assembling peptide (SAP) hydrogel. (**A**) Linear, chemical and three-dimensional structure of the (RADA)_4_-SDKP as a precursor of the SAP hydrogel. (**B**) HPLC graph related to (RADA)_4_-SDKP. (**C**) Visual illustration of gel formation by addition of Ca²⁺ to the 0.25% (RADA)_4_-SDKP solution. (**D**) Field emission scanning electron microscope (FESEM) images of 0.25% (RADA)_4_-SDKP hydrogel. (**E**) Viscoelasticity analysis of the 0.25% (RADA)_4_-SDKP hydrogel using shear rheometry. (**F**) MTS assay to test the cellular activity of neonatal mouse cardiomyocytes (NMCMs) after 1, 2, and 3 days of encapsulation in the (RADA)_4_-SDKP. MTS assay was performed in 3 biological replicates, each replicate included 4 repeats. Data are shown as mean ± standard deviation.

RADA16-I, as the core of the self-assembly, was decorated with the biologically active motif SDKP to create (RADA)4-SDKP as the precursor of a bioactive SAP hydrogel (Figure 1A). Regarding the HPLC assay that has been performed by DG Peptide company, a sharp single peak indicative of a highly pure product was observed (Figure 1B). The peptide solution was converted to hydrogel upon mixing with divalent calcium ions for less than 1 min (Figure 1C). Scanning electron microscopy confirmed the SAP hydrogel microstructure. The presence of a nanofiber-like pattern of the SAP could have been the result of binding and alignment of the peptide sequences following treatment with the positively charged ions (Figure 1D). We evaluated the rheological features of the hydrogel to investigate the changes in storage modulus (G′) and loss modulus (G″) with shear frequency as the elastic and viscous terms of gel viscoelasticity, respectively. The prominent elastic behavior indicated by a higher G’ value versus G” and the plateau behavior of both G’ and G” confirmed the successful sol–gel transition (Figure 1E). The G′ and G″ measurements indicated that a highly soft hydrogel was achieved, which guaranteed its injectability. To investigate the cell encapsulation potential of the SAP hydrogel, NMCMs were mixed with the (RADA)4-SDKP solution followed by calcium ion addition to enable the mixture to gel. The metabolic activity of the encapsulated NMCMs within the (RADA)4-SDKP hydrogel was assessed by MTS assay. The results showed no substantial change in cellular activity of the encapsulated cells compared to the cells cultured on tissue culture plate (TCP) as a control after 24 h, 48 h, and 72 h, which showed that the self-assembly process was safe for cell encapsulation and that the (RADA)4-SDKP hydrogel (0.25% concentration) was biocompatible (Figure 1F).

### 3.2. Angiogenic Potency of (RADA)4-SDKP

**Figure 2:**
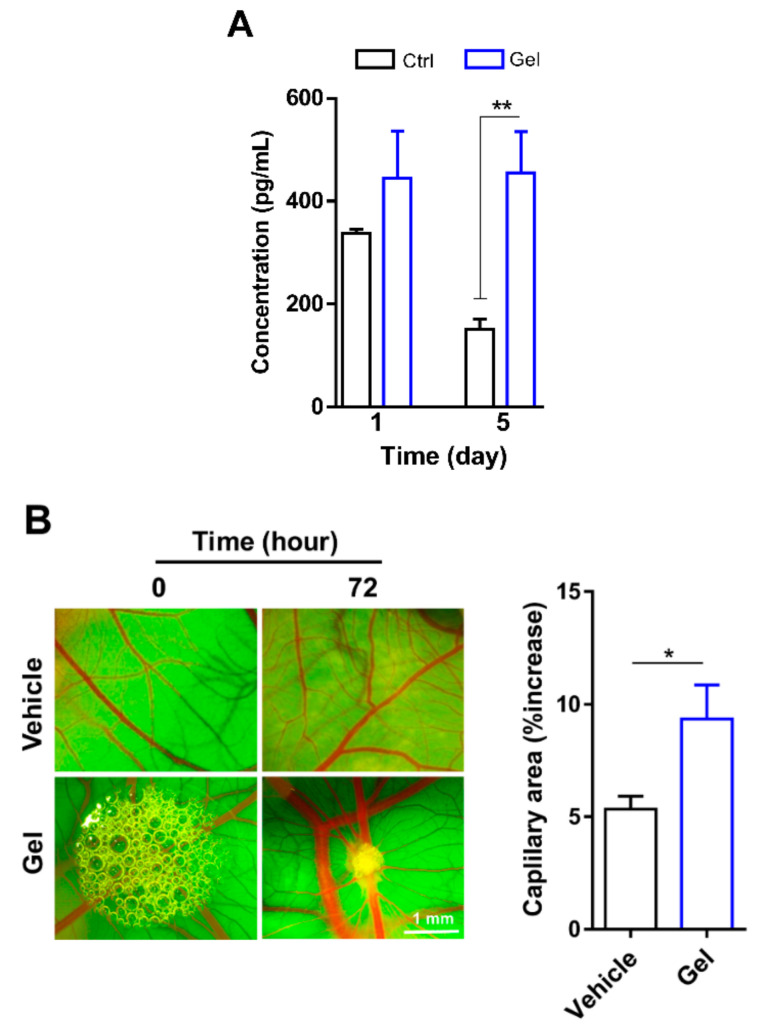
Angiogenic potential of (RADA)_4_-SDKP hydrogel. (**A**) Vascular endothelial growth factor (VEGF) release by human umbilical vein endothelial cells (HUVECs) cultured on either tissue culture plate as the control (Ctrl) or encapsulated in 0.25% (RADA)_4_-SDKP (Gel) after 1 and 5 days. (**B**) Representative images of the 0.25% (RADA)_4_-SDKP hydrogel placed on the chorioallantoic membranes (CAMs) at 0 and 72 h of incubation (*n* = 5, *p* ≤ 0.05). Quantification of blood vessel area demonstrated that the 0.25% (RADA)_4_-SDKP hydrogel resulted in a statistically significant increase in response. Data are shown as mean ± standard deviation (*n* = 3). * *p* < 0.05, ** *p* < 0.01.

The angiogenic potential of the (RADA)4-SDKP hydrogel was assessed by measurement of VEGF release and CAM assay. While VEGF release did not substantially differ between HUVECs plated on TCP (338 ± 6.9 pg/mL) and those encapsulated in hydrogel (444 ± 92.2 pg/mL) after 24 h (*p* > 0.05), the five-day culture in (RADA)4-SDKP scaffold resulted in markedly increased VEGF release compared to the amount released by HUVECs cultured on TCP (455 ± 79.9 vs. 150 ± 19.8 pg/mL, respectively, *p* ≤ 0.05; Figure 2A).

CAM is a popular angiogenesis model that is a highly vascularized membrane located beneath an egg shell. Its location provides easy access for the application of diverse substances and monitoring vessel growth in response to these substances in real time. (RADA)4-SDKP treatment for 72 h substantially increased vascular density distal to the region of the application compared to the vehicle, which indicated its angiogenic potency (9.33 ± 1.5% vs. 5.33 ± 0.5%, *p* ≤ 0.05; Figure 2B).

### 3.3. Cardiac Functional Restoration by (RADA)4-SDKP

**Figure 3:**
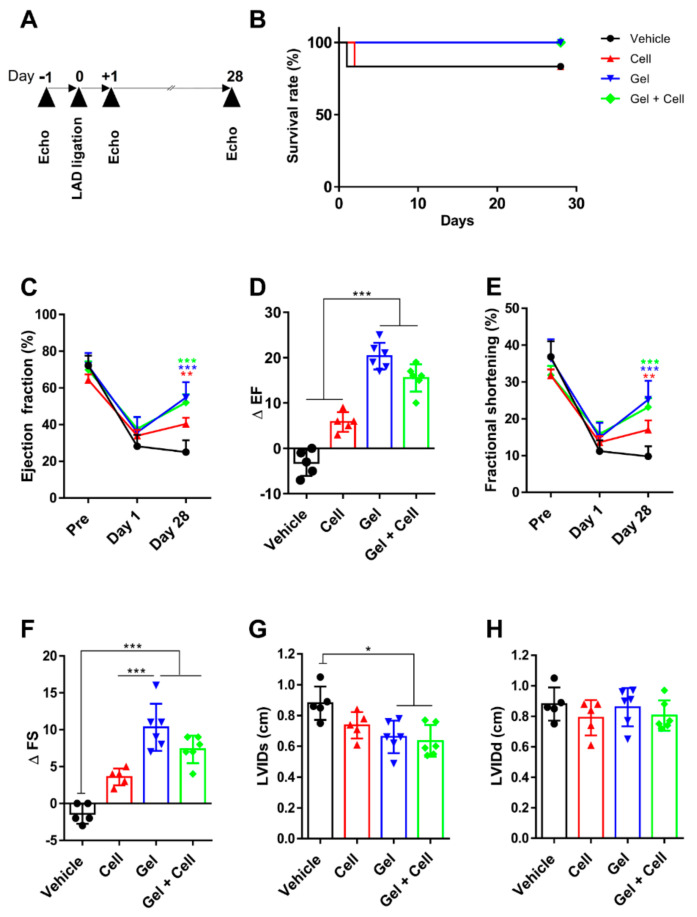
(RADA)_4_-SDKP hydrogel improved cardiac function after myocardial infarction (MI). (**A**) Schematic representation of the in vivo study design. The study animal groups included phosphate-buffered saline (PBS) as the Vehicle group, 2 × 10^6^ bone marrow-derived mesenchymal stem cells (BM-MSCs) as the Cell group, 0.25% (RADA)4-SDKP (Gel), and 2 × 10^6^ MSCs encapsulated within 0.25% (RADA)_4_-SDKP (Gel + Cell). We injected 100 µL of the individual treatments into the peri-infarct region of the rats’ hearts 15 min after induction of an acute myocardial infarction (MI). Next, echocardiographs were taken at 1 day before MI, 1 day after MI and 28 days after MI followed by histomorphometric evaluations of the rats’ hearts. (**B**) Kaplan–Meier plot demonstrated increased survival in the Gel and Gel+Cell groups compared with the Cell and Vehicle groups. (**C**) Ejection fraction (EF) in all groups at pre-operation, and days 1 and 28 after MI. At day 28, EF was increased in all of the treated groups compared with the Vehicle group. (**D**) Variations in EF from day 1 to day 28 (∆EF) was higher in the Gel and Gel + Cell groups. (**E**) Fractional shortening (FS) in all groups at pre-operation, days 1 and 28 after myocardial infarction are illustrated. On day 28, FS was higher in all treated groups compared with Vehicle. (**F**) Variation of fractional shortening from day 28 to day 1 (∆FS) was greater in Gel and Gel + Cell group. (**G**) Slight improvement was discernable in the parameter of the left ventricular inner diameter at systole (LVIDs) for the Gel and Gel + Cell groups compared with the Vehicle group. (**H**) Left ventricular inner diameter at diastole (LVIDd) did not differ significantly between groups. All data are presented as mean ± standard deviation, *n* ≥ 5, * *p* < 0.05; ** *p* < 0.01; *** *p* < 0.005.

In an attempt to investigate the potential of the (RADA)4-SDKP hydrogel for cell delivery as treatment for MI, we transplanted BM-MSCs by an intramyocardial injection and using the (RADA)4-SDKP hydrogel as the carrier. Cardiac performance was assessed by echocardiography for all groups (Vehicle, Cell, Gel, and Gel + Cell) 24 h before MI (Baseline), 1 day after MI induction (Post 1) and 28 days after MI induction (Post 2) (Figure 3A). Animal survival did not significantly differ between all groups (Kaplan–Meier; Figure 3B). Echocardiographic measurements at baseline showed normal cardiac function in selected animals (EF >70%, *p* > 0.05). After LAD ligation, the color of the cardiac muscle became white from the point of the suture down to the apex, which indicated perfusion disturbances and hypoxia. The ventricular ejection fraction was drastically reduced one day after MI induction in all groups (EF <40%, *p* > 0.05). Of note, animals with post 1 EF >40% were excluded from the experiment.

Four weeks of treatment with the Gel and Gel+Cell restored the LVEF to a large extent, which was superior to that of the Cell and Vehicle groups as seen by the ΔEF of 20.33 ± 2.94 (Gel), 15.5 ± 3.01 (Gel + Cell), 5.8 ± 2.16 (Cell), and −3.2 ± 2.86 (Vehicle) with a *p*-value of <0.001 (Figure 3C,D).

Consistent with the EF findings, contractile function of the LV showed improvement in the Gel and Gel + Cell treatment groups compared to the Vehicle group, as shown by the ΔFS of 10.33 ± 2.02 (Gel) and 7.33 ± 1.86 (Gel + Cell), *p* < 0.001 (Figure 3E,F). However, MSC transplantation did not markedly restore ventricular contractility (ΔFS: 3.6 ± 1.1 (Cell) vs. −1 ± 1.34 (Vehicle); Figure 3E,F). The LVIDs and LVIDd, which provide valuable information on contractile function of the LV and are used as indexes of ventricular wall dilation progression, were analyzed in the treatment groups. There were substantially smaller LVIDs in the Gel and Gel + Cell groups compared to the Cell and Vehicle groups (*p* < 0.05, Figure 3G). However, LVIDd did not differ among all treatment groups (*p* > 0.05, Figure 3H).

**Figure 4:**
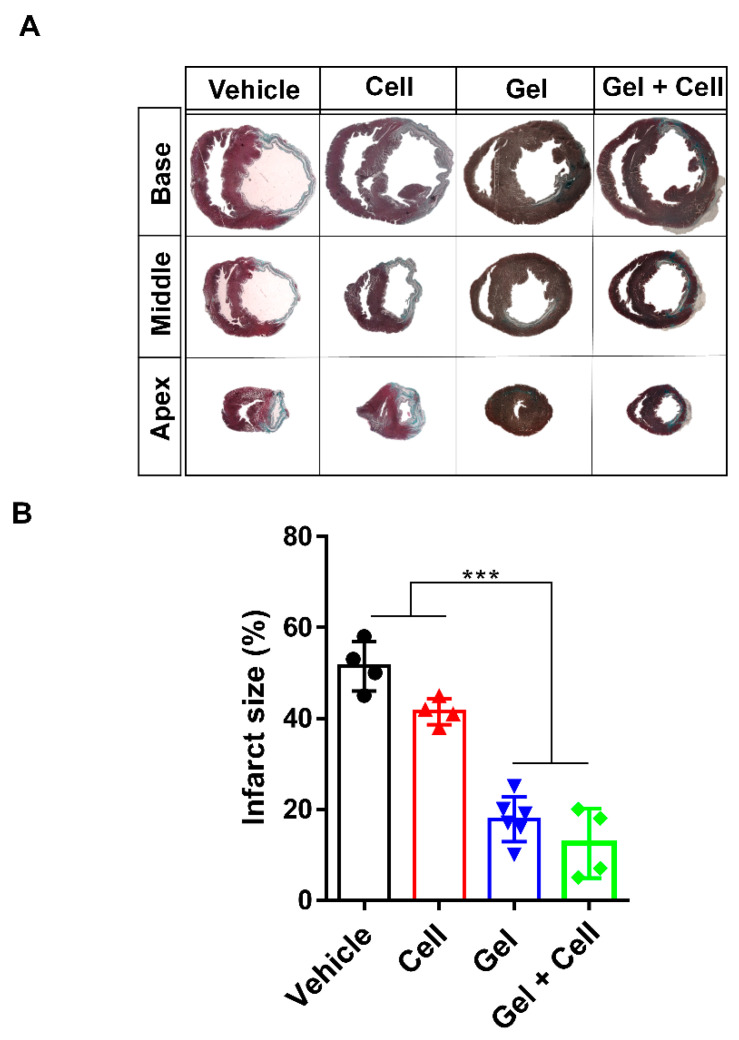
(RADA)_4_-SDKP hydrogel diminished scar size. (**A**) Representative images of Masson’s trichrome (MT) stained heart sections at the apex, middle, and base areas for all groups. (**B**) At day 28, the infarct area (% of left ventricle (LV)) was decreased in the Gel and Gel + Cell groups in comparison with the Cell and Vehicle groups. All data are presented as mean ± standard deviation (*n* ≥ 4). *** *p* < 0.001.

Quantification of the fibrotic area showed that functional improvement coincided with smaller scar formation in the infarct area of the rat hearts in the Gel (17.83 ± 4.9%) and Gel + Cell (12.5 ± 7.59%) groups compared with the Cell (41.5 ± 2.88%) and Vehicle (51.5 ± 5.44%) groups (*p* < 0.001, Figure 4A,B).

These data revealed substantial restoration of cardiac function when infarcted rat hearts were subjected to the Gel and Gel + Cell treatments. However, there appeared to be no marked differences between the Gel and Gel + Cell treatments with respect to their functional improvements, which highlighted the efficacy of the designated SAP hydrogel for cell-free cardiac functional restoration.

### 3.4. Cardioprotective Mechanisms of (RADA)4-SDKP in Infarcted Rat Hearts

**Figure 5:**
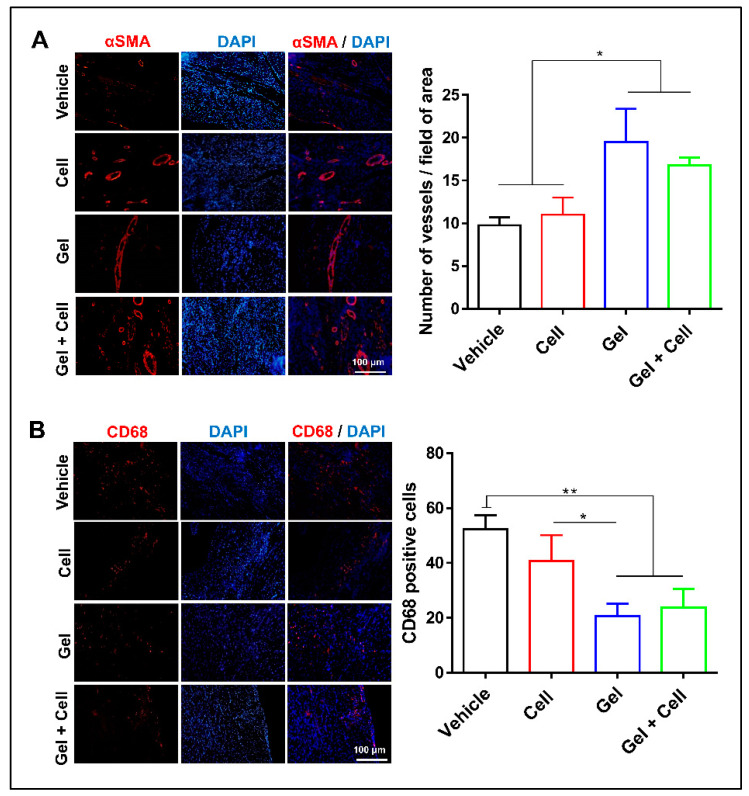
(RADA)_4_-SDKP hydrogel increased angiogenesis and reduced inflammation. (**A**) More vessels (α-SMA⁺ cells) were detected in the Gel and Gel + Cell groups compared with the Vehicle group. (**B**) A lower number of CD68⁺ macrophages were observed in the Gel and Gel + Cell groups compared with the Cell and Vehicle groups. All data are presented as mean ± standard deviation (*n* ≥ 3). * *p* < 0.05; ** *p* < 0.01.

**Figure 6:**
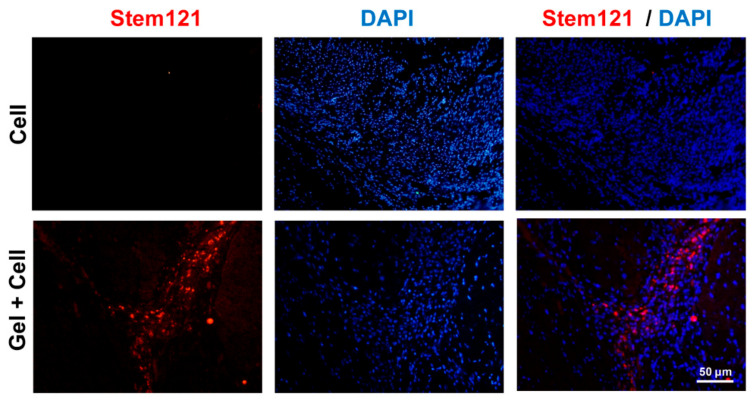
Cell retention after hydrogel-mediated delivery. At the fourth week, encapsulated mesenchymal stem cells (MSCs) that were injected into the myocardium were detected by immunostaining of STEM121 at the injection site.

(RADA)4-SDKP treatment could efficiently inhibit the failure in cardiac functional reserve after an MI, which we further investigated with respect to angiogenesis and inflammatory response in the infarcted heart. Presence of SAP hydrogel in echocardiography images of post1 was confirmed (Figure S1 and Supplementary Movie 1). However, it was absent in echocardiography images of post2 (Figure S1) and all histological sections. Thus, we concluded that the hydrogel degraded completely and disappeared after four weeks. Immunostaining by α-SMA showed a substantially larger number of vessels in the infarct area of the LV following treatment with both the Gel (19 ± 3) and Gel + Cell (16 ± 1) groups compared to the Vehicle group (9 ± 1, *p* < 0.05, Figure 5A). Consistent with in vitro and CAM assays, (RADA)4-SDKP hydrogel augmented vascularization, which might underlie cardiac functional restoration following the MI in the rats. We assessed the inflammatory response at the infarct area by CD68 immunostaining as a marker of macrophages. There were considerably fewer CD68-stained cells in the infarct areas of the hearts that received (RADA)4-SDKP hydrogel (20 ± 4) or Gel + Cell (23 ± 6) compared to the Cell (40 ± 9) or Vehicle (52 ± 5) groups (*p* < 0.05, Figure 5B). This finding indicated the probability of controlling the inflammatory response by this modified SAP hydrogel, which was comparable with the anti-macrophage recruitment of MSCs in our experimental setting. In order to check for BM-MSC retention after transplantation, STEM121 immunostaining was performed to specifically identify human cells at the injection site. The results showed the presence of STEM121^+^ cells when BM-MSCs were delivered by the (RADA)4-SDKP hydrogel (Figure 6).

## 4. Discussion

Adverse LV remodeling after MI directs cardiac tissue to a failing muscle, develops heart failure, and increases mortality [47,48]. Several strategies have been investigated for cardiac protection following MI, with over 6400 articles published since 1975; however, there is no ideal treatment available for this purpose [8].

Regenerative therapy for ischemic heart diseases, including cell-based therapy and cell-free approaches such as biomaterial scaffolds and delivery of bioactive molecules, is an active field of research that has launched several potential treatment strategies [49].

The current study examined a novel hypothesis that SDKP-conjugated SAP hydrogel would decelerate LV remodeling after AMI induction in rat hearts. This hydrogel could restore cardiac function. Furthermore, the (RADA)4-SDKP hydrogel had the capability to deliver and retain BM-MSCs at the site of the infarct. Thus, we designed a bioactive hydrogel that, in addition to an inherent cardioprotective effect, could efficiently deliver cells and improve their retention. However, the appropriate cell source should be further determined.

The SAP hydrogel has shown tremendous promise as a therapeutic biomaterial and addressed numerous indispensable requirements for myocardial therapy and regeneration [30]. RADA is a class of SAPs that can form supramolecular hydrogels upon mixing with the positively charged multivalent ions. The RADA-based hydrogels are biocompatible and biodegradable, mimic the nanofibrous structure of ECM, induce cell recruitment, and allow for sequence modification to render other bioactive functions [50,51]. These hydrogels have been used for the engineering of various tissues with low immunogenicity [52,53,54]. After injection of RADA16-II into the infarcted myocardium, the empty space of the hydrogel was cellularized by endothelial, smooth muscle, and cardiac cells within days, which showed the cell recruitment potential of the RADA-based hydrogels for endogenous regeneration of infarcted myocardium [30,55]. In order to improve cardiac function, cardioprotective and angiogenic cues such as IGF1 [56], PDGF [54], VEGF [57], FGF [58], Notch1 [16], and QHREDGS [59] have been integrated with the SAP hydrogels.

Angiogenesis is a vital mechanism that may alleviate heart dysfunction after MI. Hence, one of the key points in the regeneration of infarcted myocardium, either by resident progenitor cells or cell therapy, is sufficient angiogenesis in the target zone. Formation of new vessels and an adequate blood supply to the affected area reduces cardiomyocyte apoptosis and the level of fibrosis [34,60,61]. Some studies support the idea of SAP hydrogels modified with angiogenic motifs that promote cardiac repair. One proposed mechanism was an increase in vascular density and vessel diameter [57,61]. *N*-acetyl-seryl-aspartyl-lysyl-proline (Ac-SDKP) is a tetrapeptide that has been well studied for improvement of the adverse effects of MI. Suggested mechanisms for cardiac healing of SDKP include enhancement of angiogenesis and reduction of fibrosis and inflammation [33,34,35,36,37]. Upon addition of calcium ions to the (RADA)4-SDKP solution, the amino acids reorganize in a nanofiber-like structure to form a supramolecular hydrogel without any chemical reaction. The safe conditions of the physical crosslinking could allow for encapsulation of the viable cells during gel formation. Moreover, the nanofibrous morphology might provide an ECM-mimetic microenvironment to support functionality of the cells [31].

From the various cell sources, MSCs appear to be promising for transplantation in MI [47,62]. Encapsulation of MSCs in SAP hydrogels has been proposed to overcome low engraftment of the cells and appeared to be beneficial in preclinical studies [15,63]. Consistent with previous studies, our SAP hydrogel was highly soft (G’~60 Pa), which might provide enough flexibility to not disrupt under heart beating [16]. In addition, the low density of the physical crosslinks could allow for easy injection of the gel due to its shear-thinning potential throughout the needle. In vitro and ex ovo assessments have shown the capability of the (RADA)4-SDKP hydrogel to induce angiogenic responses. Myocardial injection of the (RADA)4-SDKP hydrogel (Gel) at the infarcted area restored cardiac function after four weeks of follow-up. The higher probability of infarct area reduction, blood vessel density increment, and inflammatory response alleviation might be attributed to inherent properties of the SDKP motif, which is distributed in the entire structure of the hydrogel. However, this should be further investigated. Particularly, it is of great value to investigate the three phases of post-infarction inflammatory response [64] to address the time-dependent activation of macrophages. These results agreed with other studies that have used SAPs in a cell-free approach [16,61,65]. We also examined the potential of using the SAP hydrogel as a cell delivery vehicle. To this end, BM-MSCs were encapsulated within the (RADA)4-SDKP hydrogel during gel formation to make a cocktail with potential cardioprotective effect (Gel + Cell group). Of note, free injection of BM-MSCs did not show restoration of cardiac function compared with the vehicle group. These data were expected due to the probability of cell washout from contracting myocardium and poor retention. Furthermore, no trace of the gel was detectable in the histological sections of 28 days’ post-treatment. This observation was in agreement with the echocardiography observations where the injected gel was clearly detectable on day 1, whereas it disappeared on day 28, suggesting complete degradation of the gel during the four-week experiment. Nevertheless, the transplanted BM-MSCs were detectable after 28 days in the infarct region when delivered by the SAP hydrogel as was confirmed by STEM121 expression, as a human cell marker.

We observed similar results on the evaluated cardiac parameters after both Gel and Gel + Cell treatments. Ichihara et al. combined SAPs and MSCs to improve engraftment via epicardial coating, which led to enhanced echocardiographic metrics of the infarcted hearts [15]. Thus, besides its inherent cardiac anti-remodeling effect following MI, (RADA)4-SDKP can be combined with an appropriate cell source to enhance the efficiency of cell therapy. In general, cell-free systems such as this modified self-assembling peptide could provide highly competitive approaches for regenerative therapy of MI.

## 5. Conclusions

Delivery of a 0.25% (RADA)_4_-SDKP hydrogel to the infarcted rat heart as a cell-free SAP-based scaffold resulted in improved cardiac function coupled with increased vessel formation and reduced fibrosis and inflammation. Intramyocardial injection of (RADA)_4_-SDKP hydrogel could be an effective acellular therapy for myocardial repair with the possibility for clinical translation.

## Supplementary Materials

The following are available online at https://www.mdpi.com/2218-273X/10/2/205/s1, Figure S1: Presence assessment of SAP hydrogel at Post1 and Post2, Movie S1: Presence assessment of SAP hydrogel at Post1.
