# Correction: Firoozi et al. A Cell-Free SDKP-Conjugated Self-Assembling Peptide Hydrogel Sufficient for Improvement of Myocardial Infarction. *Biomolecules* 2020, *10*, 205

**DOI:** 10.3390/biom14070751

**Published:** 2024-06-25

**Authors:** Saman Firoozi, Sara Pahlavan, Mohammad-Hossein Ghanian, Shahram Rabbani, Shima Tavakol, Maryam Barekat, Saeed Yakhkeshi, Elena Mahmoudi, Mansoureh Soleymani, Hossein Baharvand

**Affiliations:** 1 Department of Tissue Engineering and Regenerative Medicine, Faculty of Advanced Technologies in Medicine, Iran University of Medical Sciences, Tehran 1449614535, Iran; saman.nano1989@gmail.com; 2 Department of Stem Cells and Developmental Biology, Cell Science Research Center, Royan Institute for Stem Cell Biology and Technology, ACECR, Tehran 1665659911, Iransaeedyakhkeshi@gmail.com (S.Y.); 3 Department of Cell Engineering, Cell Science Research Center, Royan Institute for Stem Cell Biology and Technology, ACECR, Tehran 1665659911, Iran; biomaterialist@gmail.com; 4 Research Center for Advanced Technologies in Cardiovascular Medicine, Tehran Heart Center, Tehran University of Medical Sciences, Tehran 1416753955, Iran; sh-rabbani@tums.ac.ir; 5 Cellular and Molecular Research Center, Iran University of Medical Sciences, Tehran 1449614535, Iran; shima.tavakol@yahoo.com; 6 Department of Regenerative Medicine, Cell Science Research Center, Royan Institute for Stem Cell Biology and Technology, ACECR, Tehran 1665659911, Iran; barekat1001@yahoo.com; 7 Massachusetts General Hospital, Harvard Medical School, Boston, MA 02114, USA; elena.mahmoudi@gmail.com; 8 Department of Developmental Biology, University of Science and Culture, ACECR, Tehran 1461968151, Iran

**Error in Figure**

The authors wish to make the following corrections to this paper [1]. Following publication, it was discovered that some of the representative images provided in Figures 4 and 5 have overlap with that in another published paper [2] and are incorrect. This occurred because the in vivo experiments were performed simultaneously with manuscript [2]. The two studies were related and investigated the regenerative potential of hydrogel-encapsulated mesenchymal stem cells (MSC) as well as MSC-derived exosomes for rat hearts after myocardial infarction. In order to minimize the number of control ani-mals as much as possible and to reduce the number of sacrificed rats for these projects, the same control rats served as the control group for all the treatment groups in both papers. This explains why the provided bar plot for the control group (vehicle) rats appeared the same in both figures from the two articles. To avoid overlapping figures, the representative images of vehicle group in Figure 4A and Figure 5A,B. are replaced as below.

Furthermore, the representative images of Gel group were incorrect in Figure 5A and are therefore replaced.

The authors would like to apologize for any inconvenience caused to the readers of Biomolecules for this error. We would like to state that the scientific conclusions were un-affected. The published version will be updated on the article webpage, with a reference to this correction notice.

Due to an issue relating to intellectual property, the (RADA)4-SDKP peptide sequence in Materials and Methods section is deleted as the authors requested. A correction has been made to **2. Materials and Methods**, *2.1. Preparation of the (RADA)4-SDKP Hydrogel*.

## Figures and Tables

**Figure 4:**
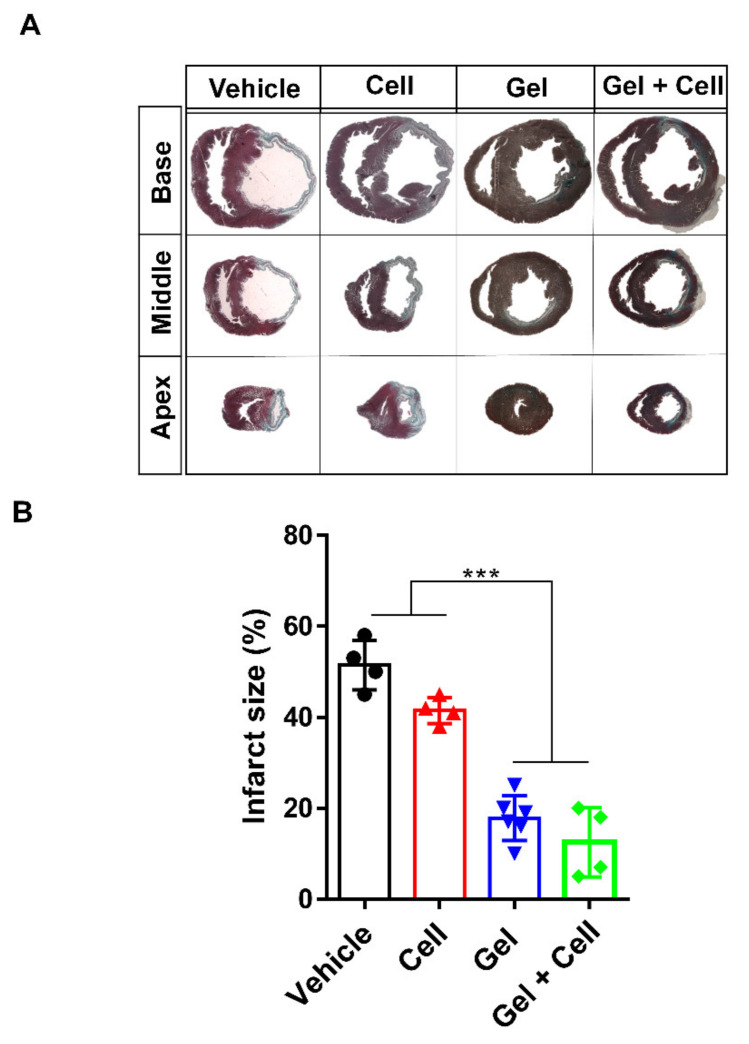
(RADA)_4_-SDKP hydrogel diminished scar size. (**A**) Representative images of Masson’s trichrome (MT) stained heart sections at the apex, middle, and base areas for all groups. (**B**) At day 28, the infarct area (% of left ventricle (LV)) was decreased in the Gel and Gel + Cell groups in comparison with the Cell and Vehicle groups. All data are presented as mean ± standard deviation (n ≥ 4). *** *p* < 0.001.

**Figure 5:**
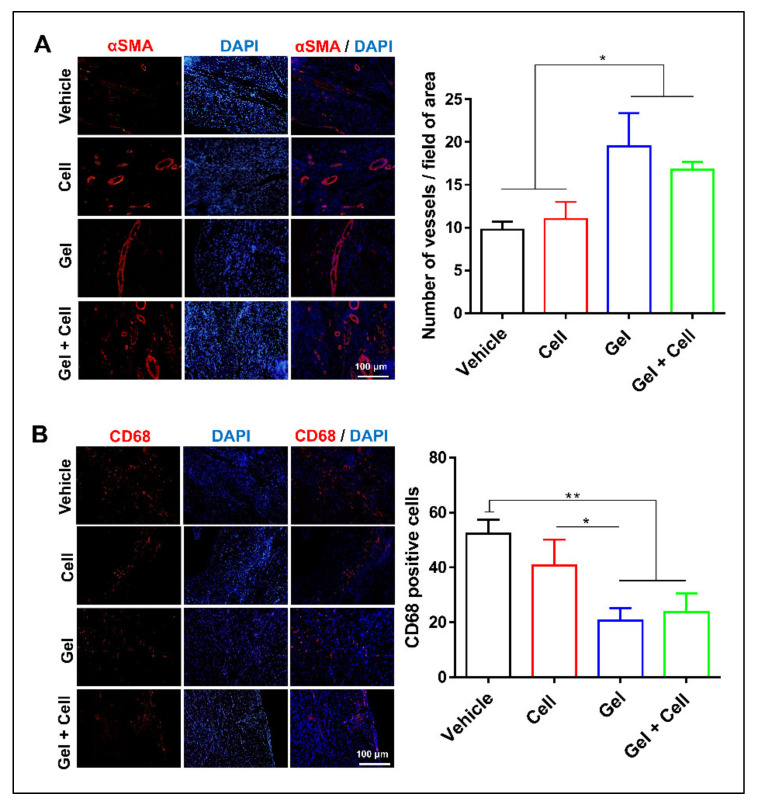
(RADA)_4_-SDKP hydrogel increased angiogenesis and reduced inflammation. (**A**) More vessels (α-SMA⁺ cells) were detected in the Gel and Gel + Cell groups compared with the Vehicle group. (**B**) A lower number of CD68⁺ macrophages were observed in the Gel and Gel + Cell groups compared with the Cell and Vehicle groups. All data are presented as mean ± standard deviation (n ≥ 3). * *p* < 0.05; ** *p* < 0.01.
